# Feeding Tall Fescue Seed Reduces Ewe Milk Production, Lamb Birth Weight and Pre-Weaning Growth Rate

**DOI:** 10.3390/ani10122291

**Published:** 2020-12-03

**Authors:** Jessica L. Britt, Maslyn A. Greene, Sarah A. Wilbanks, J. Keith Bertrand, James L. Klotz, William Bridges, Glen Aiken, John G. Andrae, Susan K. Duckett

**Affiliations:** 1 Department of Animal and Veterinary Sciences, Clemson University, Clemson, SC 29634, USA; jlbritt@clemson.edu (J.L.B.); maslyng@clemson.edu (M.A.G.); adams9@clemson.edu (S.A.W.); jkbertr@clemson.edu (J.K.B.); 2 USDA-ARS, Forage Production Research Unit, Lexington, KY 40546, USA; James.klotz@usda.gov; 3 Department of Mathematical Sciences, Clemson University, Clemson, SC 29634, USA; wbrdgs@clemson.edu; 4 North Florida Research and Education Center, University of Florida, Quincy, FL 32351, USA; gaiken@ufl.edu; 5 Department of Plant and Environmental Sciences, Clemson University, Clemson, SC 29634, USA; jandrae@clemson.edu

**Keywords:** sheep, tall fescue, ergot alkaloids, vasoconstriction, milk, weaning

## Abstract

**Simple Summary:**

This study was conducted to examine how ergovaline/ergovalinine ingestion during pregnancy in ewes with different *DRD2* genotypes altered ewe and lamb performance. Feeding ergovaline/ergovalinine in last trimester reduced lamb birth weight; however, milk production was lower for all ewes fed ergovaline/ergovalinine. Lambs born to dams fed ergovaline/ergovalinine had slower growth rates and lower weaning weights. These results demonstrate that ingestion of ergot alkaloids during pregnancy can negatively influence lactation, growth of offspring and birth and weaning weights. Recommendations would include strategies to mitigate mycotoxin exposure in pregnant ewes to improve production.

**Abstract:**

Endophyte-infected tall fescue (E+) produces ergovaline and ergovalinine, which are mycotoxins that act as dopamine agonists to suppress prolactin and induce vasoconstriction. The experiment was designed as a 3 × 2 × 2 factorial with *DRD2* genotype (AA, AG, GG), fescue seed (endophyte-free, E− or endophyte-infected, E+), stage of gestation (MID, d (day) 35–85; LATE, d 86–parturition) and all interactions in the model. Pregnant Suffolk ewes (n = 60) were stratified by genotype and fed E+ or E− seed in a total mixed ration according to treatment assignment. Serum prolactin concentrations were lower (*p* < 0.05) in ewes fed E+ seed but did not differ by maternal *DRD2* genotype or two-way interaction. Lamb birth weight was lower (*p* < 0.05) in ewes fed E+ seed in last trimester. Pre-weaning growth rate, milk production and total weaning weight was reduced (*p* < 0.05) in ewes fed E+ fescue seed during MID and LATE gestation. Ingestion of ergovaline/ergovalinine in last trimester reduces lamb birth weight; however, lamb growth rate, milk production and total weaning weight are reduced in all ewes fed E+ during mid and last trimester.

## 1. Introduction

In the Eastern U.S., the main cool season perennial forage available for grazing ewes during gestation is tall fescue (*Lolium arundinaceum* (Schreb.) Darbysh; *Schedonorus phoenix* (Scop.) Holub). Animals grazing tall fescue have reduced body weight gains [1,2] and reproductive problems [3,4]. Poor animal performance is related to ingestion of ergot alkaloids, a class of mycotoxins, produced by the endophyte (*Epichloë coenophiala*) [5]. Maternal consumption of ergopeptines like ergovaline reduces birth weights in calves [6] and lambs [7,8]. Further research suggests that reductions in offspring birth weights are due to intrauterine growth restriction (IUGR) [9] resulting from ergot alkaloid-induced vasoconstriction [10].

Ergopeptine alkaloids, especially ergovaline and its epimer ergovalinine, are D2 agonists that bind to dopaminergic receptors and suppress serum prolactin concentrations [11,12,13]. The structure of ergopeptines is comparable to serotonin and dopamine [14,15,16] and they act as agonists, resulting in prolactin suppression and vasoconstriction [17,18]. Many researchers have shown that ingestion of ergot alkaloid lowers serum prolactin, which is critical for mammary development and milk production [19,20,21,22]. For these reasons, the dopamine receptor D2 (*DRD2*) gene was selected as a candidate to investigate a possible genetic tolerance with fescue toxicosis. In cattle, a Single Nucleotide Polymorphism (SNP) in the DRD2 gene was related to changes in prolactin response to tall fescue [23]. The objectives of this study were to: (1) determine if the DRD2 genotype is associated with response to E+ fescue seed treatment, and (2) examine how feeding tall fescue seed containing ergovaline/ergovalinine in second and third trimester modifies ewe performance, lactation and growth of offspring.

## 2. Materials and Methods

Clemson University Institutional Animal Care and Use Committee approved the use of animals in this experiment (AUP 2014-081).

### 2.1. Experimental Design

This is a follow-up study to our previous work [8] in which the same experimental design and seed feeding methodologies to induce fescue toxicosis at specific stages of gestation was used. The difference with this study was that all ewes went to term in order to measure birth weight, milk production and pre-weaning growth rates. Suffolk ewes (n = 83) were obtained from four farms and were naïve to tall fescue. Ewes were genotyped for the dopamine receptor D2 (*DRD2*). Pregnant ewes (n = 60) were stratified by genotype (AA, AG, GG) and randomly assigned to fescue seed treatments (E− or E+) and stage of gestation (MID, d 35–85 and/or LATE, d 86–parturition). This design provides four fescues by stage of gestation treatment, E−/E-, E−/E+, E+/E− and E+/E+, where “/” denotes mid/late gestation fescue treatment. Tall fescue seed was mixed daily into a total mixed ration (TMR; 35% corn, 25% cottonseed hulls, 20.5% soybean hulls, 14% molasses, 4.5% soybean meal and 1% limestone) and individually fed to each ewe according to treatment. Nutrient analyses were performed on seed and TMR samples, and rations were formulated to meet National Research Council (NRC) requirements [24] for gestating ewes from early (d 35–85) and late (d 86–parturition) gestation. Ewes receiving E+ fescue treatment were fed E+ seed at a level to provide 1.77 mg/hd/d of ergovaline/ergovalinine. The dose level of ergovaline/ergovalinine utilized was based on previous experiments [7,8,25]. Seed lots were sampled and analyzed for ergovaline, ergovalinine and ergotamine content according to Aiken et al. [25]. All seed cultivars tested negative for ergotamine. Ewes receiving the E− fescue treatment were fed the same amount of E− seed that contained 0 mg/hd/d of ergovaline/ergovalinine. Ewes were individually stalled for feeding and any remaining orts were weighed to determine actual dry matter intake (DMI) for each individual ewe. Ewes were ultrasounded transrectally at d 30 of gestation to confirm pregnancy (Aloka 500 V) and transabdominally at d 45 to 60 of gestation (BCF Easi-Scan Portable Ultrasound Machine, BCF Technology Ltd., Rochester, MN, USA) to confirm maintenance of pregnancy and estimate lamb number. Additional details on experimental design, ewe synchronization methods, TMR nutrient composition and seed feeding rationale are available in Britt et al. [8].

### 2.2. Ewe Genotyping

Liver tissue was collected after euthanasia of ewes (n = 32) from a previous, terminal experiment with the same fescue seed treatment and stage of gestation design [8]. The DNeasy Blood and Tissue Kit from Qiagen (Germantown, MD, USA) was used to extract DNA. The SNP discovery and genotyping assays were conducted at University of Tennessee. Primers were designed to amplify overlapping sequences within the ovine *DRD2* coding sequence. Ten SNPs in the *DRD2* gene were identified; however, only one SNP had genotypic frequencies that were distributed among the three possible genotypes (NC_040266.1:26511792-26512187) and was further investigated. The discovered SNP was located in the ovine DRD2 gene in exon 1 at position 270 with a guanine/adenine substitution. A TaqMan genotyping assay (Applied Biosystems) used for genotyping and the DRD2 genotype frequency was 21% AA, 50% AG and 29% GG.

### 2.3. Blood Samples

On d 35, 55, 85, 110 and 133 of gestation and at parturition, blood samples were obtained by jugular venipuncture into serum or ethylenediaminetetraacetate (EDTA) tubes. Plasma tubes were immediately placed on ice and serum tubes were allowed to clot. Tubes were then centrifuged at 537× *g* for 20 min at 4 °C and samples stored at −20 °C for later analysis. At these time periods, the weight of each ewe also was obtained. Serum prolactin concentrations were measured via radioimmunoassay (RIA) [26] with an inter-assay and intra-assay variance of 6.19% and 6.53%.

A subset of ewes (n = 26) carrying twins that represented all fescue seed treatments by stage of gestation and maternal genotypes were selected for blood hormone metabolite analyses to assess insulin sensitivity. The subset of ewes with twin lambs was selected in order to also obtain serum metabolite measurements on a male lamb from each of these ewes during postnatal growth. Plasma glucose concentrations were analyzed using a colorimetric assay (Glucose Hexokinase Reagent, Pointe Scientific, Canton, MI, USA) with an inter-assay and intra-assay variance of 3.2% and 9.9%, respectively. Serum non-esterified fatty acid (NEFA) levels were measured using an enzyme-linked immunosorbent assay (ELISA) kit (MaxDiscovery NEFA ELISA kit 5620-01, Perkin Elmer, Austin, TX, USA) with an inter-assay and intra-assay variance of 9.9% and 9.7%. Serum NEFA ELISA was validated by comparison to chloroform–methanol extraction and GLC analyses of non-esterified, free fatty acids. Plasma insulin concentrations were measured using an ovine insulin ELISA kit (Mercodia, NC, USA) with an inter-assay and intra-assay variance of 6.8% and 8.5%, respectively. Plasma glucose and insulin assays were validated for use in ovine samples previously [27]. For each ewe, the Revised Quantitative Insulin Sensitivity Check Index (RQUICKI) was calculated [28].

### 2.4. Doppler Ultrasound

Doppler ultrasound measurements [25] on the carotid artery of each ewe were collected at one time point in LATE gestation (d 123 ± 7). Ewes were ultrasounded in their individual feeding stalls after consumption of TMR and seed to reduce excitability. Ewes were shaved at the ultrasound site one day prior to ultrasound. Color Doppler ultrasound images of left carotid artery cross-sections were collected using a Classic Medical TeraVet 3000 Ultrasound Unit (Classic Universal Ultrasound, Tequesta, FL, USA) with a 12L5-VET (12 MHz) linear array transducer. Scan depth was set at 4 cm. Cross-sectional images were collected for each artery using a frequency of 5.0 MHz and a pulse repetitive frequency that ranged between 2.5 and 3.0 kHz. Following freezing of an individual scan, frames stored in the cine memory of the unit were searched to store the image exhibiting the maximum flow signal, assumed to be at peak systolic phase. The flow signal was traced to estimate lumen area [24]. Ewes were ultrasounded in their individual feeding stalls after consumption of TMR and seed. Gestation d was recorded for each ewe and included as a covariate in the model.

### 2.5. Lambing and Milk Production

On d 133 of gestation, rectal temperature was measured in each ewe in the individual stall prior to feeding. Beginning at approximately d 135 of gestation, ewes were monitored every half hour for signs of lambing. Assistance was given if ewes had not progressed sufficiently within 45 min of the amniotic sac rupturing. Lambing difficulty and lamb vigor scores were recorded according to the procedures of Matheson et al. [29]. Lambs were weighed immediately prior to suckling and then tagged. Birth weight and sex of each lamb was recorded. Five ewes had to be removed from the study due to major complications. Two ewes had pregnancy toxemia (both E−/E−), one ewe had respiratory disease (E+/E−), and two ewes had uterine prolapses (E+/E− and E+/E+) that all required veterinary treatment or euthanasia. One ewe did not lamb according to the estimated breeding dates and therefore was removed from the study due to incorrect assignment of treatment and feeding level according to actual gestation times (E−/E+). Two ewes were confirmed pregnant by ultrasound but never lambed (E−/E+ and E+/E+) and were removed from the dataset.

After parturition (approximately d 145), all ewes were fed TMR without any seed. Udder measurements included width and circumference at the widest part of the udder and the length of each teat, which was averaged [30]. Ewes and their lambs were individually stalled in lambing jugs (8.85 m^2^) for 48 h following parturition to facilitate bonding and estimate milk production. Ewes were placed on a two-day milk production test following parturition. At 0800, ewes were milked out by hand and a dividing panel was positioned within the lambing jug to allow nose-to-nose contact but prevent nursing. After three h, dams were milked out by hand and the milk weighed. Lambs were bottle fed the collected milk and the dividing panel was removed. To estimate peak milk production [31], this procedure was also repeated at d 21 with dams and lambs separated in different pens for the three-hour duration. Weaning of the lambs from the dams occurred at 75 ± 2 d of age.

Agalactia is a known side effect of ergot alkaloid exposure in horses [32], dairy cattle [33] and beef cattle [34]. Therefore, a protocol was in place for the management and supplementation of these lambs. During the initial 24 h after birth, lambs born to dams with agalactia (3 E+/E+ ewes; 1 E+/E− ewes) were supplemented with Bovine IgG Colostrum Replacement for lambs (Land O Lakes, MN, USA) as instructed by manufacturer. After the first 24 h, lambs from dams with agalactia were supplemented with milk replacer (Sav-A-Lam, Sav-A-Caf Products, Chilton, WI, USA). Lambs remained on milk replacer supplement until weaning. Male lambs were castrated and all lambs were tail docked within 7 d of birth.

### 2.6. Statistical Analysis

All variables were tested for normality (univariate procedure of SAS (SAS Inst. Inc., Cary, NC, USA)). Serum prolactin concentrations were not normally distributed and were log-transformed. Ewe udder characteristics and milk production values were not normally distributed and were square root transformed. Ewe was considered the experimental unit for this study. Data were analyzed using a mixed model (SAS 9.4, SAS Inst. Inc., Cary, NC, USA) as a 3 × 2 × 2 factorial with the fixed effects of maternal DRD2 SNP genotype (AA, AG, GG), fescue seed treatment (E− or E+), stage of gestation (MID or LATE) and all interactions in the model. For ewe plasma and serum analytes, repeated measures analyses with autoregressive covariance structure were used to evaluate fescue seed treatment, stage of gestation, maternal genotype, sampling day and interactions in the model. Lamb number born or raised per ewe was included as a covariate when significant (*p* < 0.05). The three-way interactions between genotype, fescue seed treatment and stage of gestation were non-significant. All two-way interactions between genotype and fescue seed treatment or stage of gestation were non-significant. A protected least significant difference procedure was used to separate means. The effects were considered significant at *p* < 0.05, with trends discussed at *p*-levels between 0.05 and 0.10.

## 3. Results

**Figure 1:**
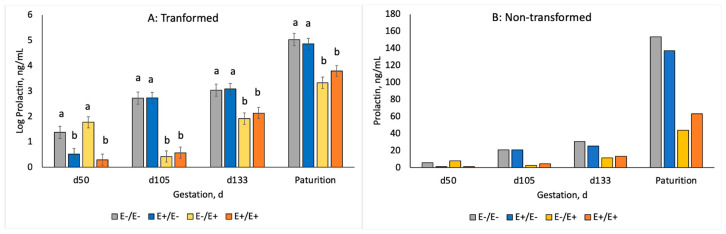
Serum prolactin concentrations, log-transformed for statistical analyses (**A**) or actual means (**B**), in ewes fed E− or E+ fescue seed from d 35 to 85 and/or d 85 to parturition. ^a,b^ Superscripts denote differences (*p* < 0.05) by day of gestation.

**Figure 2:**
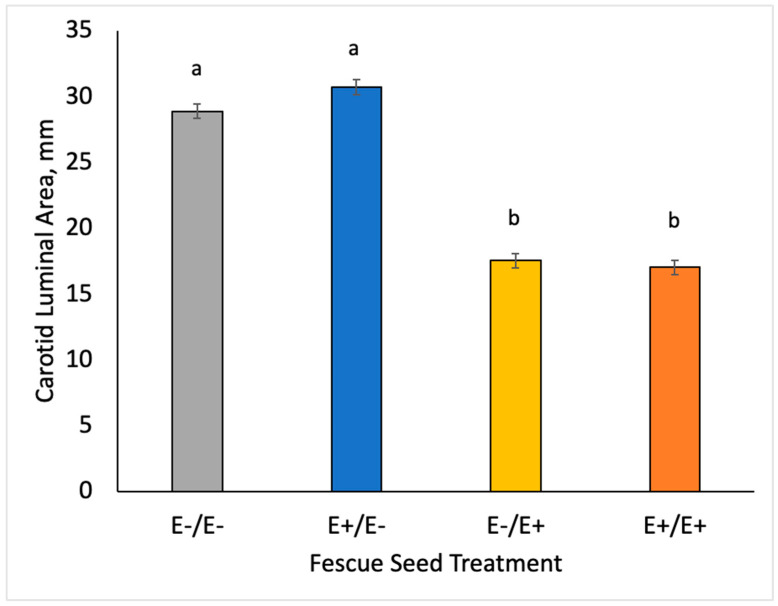
Carotid luminal area was measured on d 123 ± 7 in ewes fed E− or E+ fescue seed from d 35 to 85 and/or d 85 to parturition. ^a,b^ Superscripts denote differences (*p* < 0.05).

Non-normal variables were analyzed as transformed variables for the statistical analyses; however, results are presented as transformed means with statistical differences and actual means for easier understanding and interpretation of results. Ewe prolactin concentrations for maternal genotype and fescue seed treatment across gestation are shown in Figure 1. Ewes on E+/E− or E+/E+ treatments had lower (*p* < 0.0001) serum prolactin concentrations on d 55 than those fed E− fescue seed. Ewes on E−/E+ and E+/E+ treatments had decreased (*p* < 0.0001) serum prolactin concentrations at d 105, 133 and at parturition compared to ewes fed E− seed. Carotid luminal area was used to measure vasoconstriction at d 123 ± 7 of gestation (Figure 2). Ewes on E+/E− or E+/E+ treatments had similar carotid luminal areas to ewes fed E− seed during MID gestation. Carotid luminal area was decreased (−40%; *p* < 0.0001) in ewes fed E+ instead of E− seed at LATE gestation.

**Figure 3:**
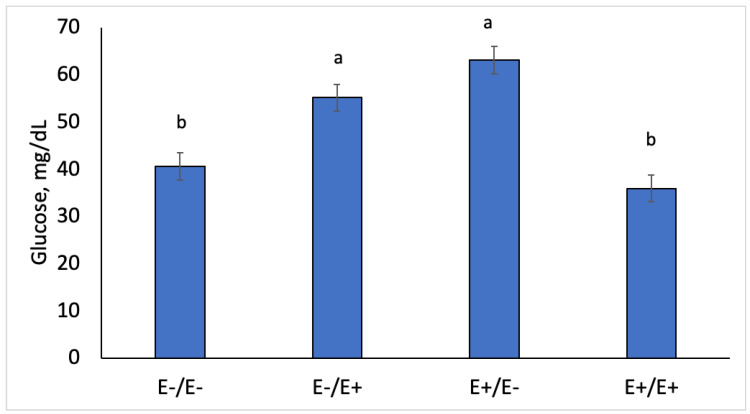
Average plasma glucose (mg/dL) concentrations during gestation in ewes fed E− or E+ seed in gestation. Superscripts denote significance (^a,b^ *p* < 0.05).

**Figure 4:**
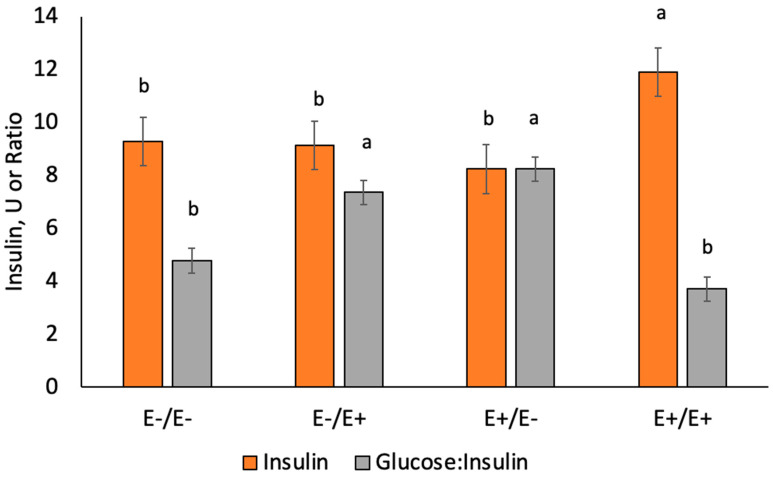
Average plasma insulin (U) concentrations and glucose to insulin ratio during gestation for ewes fed E− or E+ seed. Superscripts denote significance (^a,b^ *p* < 0.05) by variable.

Maternal metabolite concentrations were measured in a subset of ewes during gestation by maternal genotype and fescue seed treatment. The fescue seed treatment and stage of gestation interaction was significant (*p* < 0.05). Plasma glucose concentrations increased (*p* < 0.0001) in E+/E− and E−/E+ versus E−/E− and E+/E+ (Figure 3). Plasma insulin concentrations increased (*p* = 0.041) for E+/E+ compared to others (Figure 4). Plasma glucose and insulin values increased during gestation (*p* = 0.001; data not shown) but all interactions with day were non-significant. The ratio of glucose to insulin decreased (*p* < 0.0001) in E+/E+ and E−/E− compared to E+/E− and E−/E+.

**Figure 5:**
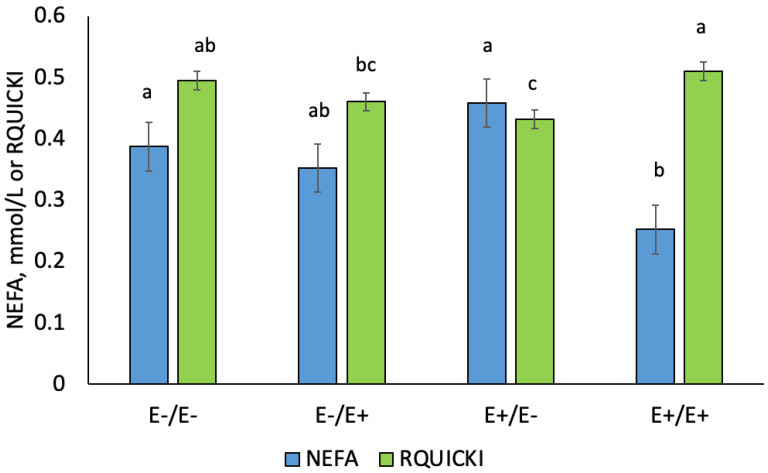
Average serum non-esterified fatty acid (NEFA; mmol/L) concentrations and RQUICKI values during gestation in ewes fed E− or E+ seed in gestation. Superscripts denote significance (^a,b.c^ *p* < 0.05) by variable.

Serum NEFA values were decreased (*p* = 0.038) for E+/E+ compared to E+/E− and E−/E− (d 85 to 133; Figure 5). Serum NEFA was highest (*p* < 0.001) on d 133 versus d 85 and 105 (data not shown). The RQUICKI values were highest (*p* = 0.0008) for E+/E+ and lowest for E+/E− across gestation. The RQUICKI values decreased (*p* < 0.0001) during LATE gestation (data not shown) but all interactions with day were non-significant.

Ewe body weight (BW) change, DMI and average daily gain (ADG) by maternal genotype, fescue seed and gestation stage are shown in Table 1. Neither maternal genotype nor fescue altered (*p* > 0.05) DMI, which was controlled in this study. Exposure to E+ fescue seed during MID gestation did not alter BW change or ADG; however, BW change and ADG were altered in LATE even when consuming the same amount of feed. Ewes fed E+ during LATE gestation had decreased BW change (*p* = 0.018) and ADG (*p* = 0.018) throughout the study (d 28 to 136) compared to those fed E−.

Parturition characteristics including gestation length, lambing difficulty, estimated lamb number per ewe, lamb number born per ewe, lamb number alive per ewe, lamb vigor scores, lamb sex ratio or crown-rump length were not different (*p* > 0.20) among fescue or genotype treatments (Table 2). Lamb birth weight (kg/ewe) did not differ when E+ fescue seed was fed during MID (d 35 to 85) gestation. Lamb birth weight (kg/ewe) was decreased (*p* = 0.02) by 10.2% in E+/E+ and E−/E+ ewes. Birth weight was strongly associated with lamb weaning weight (r = 0.81; *p* < 0.0001) in this study.

**Figure 6:**
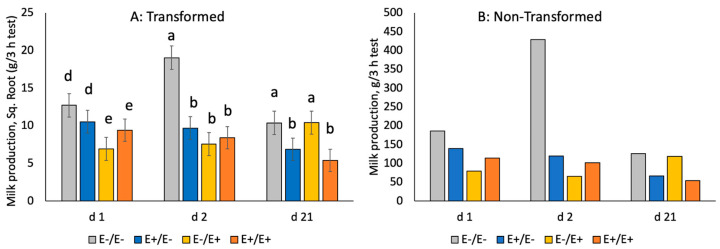
Milk production (g/3 h test) in ewes fed E− or E+ seed in MID or LATE gestation. Superscripts denote significance (^a,b^ *p* < 0.05; ^d,e^ *p* < 0.10) by day of lactation. Milk production data were not normally distributed and were square-root-transformed for statistical analyses (**A**). Actual means, non-transformed, are also presented for easier understanding and interpretation (**B**).

There was a trend for reduced (*p* = 0.091) milk production on d 1 of lactation in ewes fed E+ seed during LATE gestation (Figure 6). This reduction in milk production was associated with smaller udder circumference (*p* = 0.0087) and teat length (*p* = 0.026), whereas udder width was not altered (data not shown). On d 2 of lactation, milk production was decreased (*p* = 0.001) by 69% to 83% for all ewes fed E+ seed, regardless of stage of gestation. On d 21 of lactation, milk production was decreased (*p* = 0.028) by 51% in E+/E− and E+/E+.

Lamb BW change and average daily gain (ADG; kg/ewe) differed by maternal DRD2 genotype (Table 3). Lambs born to AG genotype ewes had greater BW change (*p* = 0.0056) and ADG (*p* = 0.010) from birth to d 28 compared to AA or GG. The change in BW tended to be greater (*p* = 0.073) and ADG was greater (*p* = 0.045) for AA compared to GG from d 28 to 56. From d 56 to weaning, lamb BW change tended to be greater (*p* = 0.085) for AG than AA but ADG was unchanged by maternal DRD2 genotype. Overall, ADG was greater (*p* = 0.0026) for AG than AA or GG. Final weaning weight per ewe was greater (*p* = 0.0039) for AG than AA or GG.

There was a trend for reduced lamb BW change from birth to d 28 (*p* = 0.083) and from d 28 to 56 (*p* = 0.053) in lambs born to E+/E− and E+/E+ ewes. Average daily gain (ADG) was decreased in E+/E− and E+/E+ lambs at birth to d 28 (*p* = 0.016) and from d 28 to 56 (*p* = 0.044). From d 56 to weaning, BW change or ADG did not differ. Overall, ADG was decreased (*p* = 0.0091) by 15% for lambs exposed to E+ at MID gestation but did not differ (*p* = 0.12) during LATE gestation. Total lamb weaning weight was reduced (*p* = 0.044) by 12.5% in E+/E− and E+/E+ ewes. There also was a trend for decreased (*p* = 0.089) total weaning weight in E−/E+ and E+/E+ ewes.

## 4. Discussion

Ergovaline is the primary ergopeptine and causative agent in fescue toxicosis [35,36,37]. Serum prolactin concentrations were reduced during the period in which ewes were fed the E+ ergot alkaloid containing seed. Many others have shown similar decreases in serum prolactin with ergot alkaloid exposure in sheep [7,8,19,38], goats [39], cattle [19,40] and horses [21]. In this study, interactions of maternal *DRD2* genotype and fescue seed treatment were non-significant (*p* > 0.15). Campbell et al. [23] discovered an SNP in bovine *DRD2* gene that was associated with serum prolactin response in cows grazing E+ fescue pastures. Our results suggest that *DRD2* SNP in the ovine may not be associated with prolactin response to E+ fescue consumption when fed as seed.

Vessel luminal area provides a direct measure of vasoconstriction in goats [39], cattle [25], sheep [40] and horses [41]. Ewes consuming E+ at the time of measurement had lower carotid luminal areas than E−. Ewes changed from E+ to E− on d 85 had similar carotid luminal areas as the controls (E−/E−), suggesting that vasoconstrictive effects from MID E+ fescue seed may be alleviated by 37 d after removal of E+ treatment. Aiken et al. [25] found that feeding E+ tall fescue seed at 0.8 mg ergovaline/kg DM constricted the caudal artery after 27 h. Similarly, Klotz et al. [42] reported that caudal arteries in cattle removed from grazing E+ fescue to drylot required 35 d to return to relaxed area similar to bermudagrass pasture controls.

Previous research [9] indicated that there may be alterations in insulin sensitivity when ergot alkaloids are fed to pregnant ewes. In this study, we evaluated insulin sensitivity using glucose to insulin ratio and RQUICKI calculations. Ewes moved from E+ to E− or vice versa on d 86 had higher glucose to insulin ratios and lower RQUICKI values compared to E+/E+ and E−/E−. Ewes that were fed E+ tall fescue seed throughout gestation had lower glucose to insulin ratio and higher RQUICKI values. A low glucose to insulin ratio and high RQUICKI value indicates greater insulin sensitivity in E+/E+ ewes [28]. Pregnancy toxemia is a condition that develops in near-term pregnant ewes related to insulin sensitivity [43,44]. In this study, we only observed pregnancy toxemia in two E−/E− ewes that had to be removed from the study and not in any of the ewes that were fed E+ fescue seed.

Differences in body weight related to E+ fescue treatment are often attributed to reduced DMI intake [34,44,45] in grazing experiments. Therefore, ewes were fed at the same level so that nutrient intake was not confounded with ergot alkaloid exposure in this study. No differences in dry matter intake were observed. Earlier studies [8] feeding fescue seed showed small reductions in DMI and ADG during MID gestation even when fed at the same level due to E+ ewes not cleaning up all feed/seed offered. Most studies that demonstrate IUGR in lambs are related to under-nutrition or over-nutrition caused by restricting or over-feeding ewes in relation to nutrient requirements during gestation [46,47,48]. Therefore, differences in ewe and offspring parameters in this study are due to mycotoxin consumption and not to nutrient intake differences.

Ergot alkaloid exposure did not alter lambing parameters. Similarly, Bond et al. [49] reported no change in gestation day, lambing rate or survival for ewes exposed to E+ fescue. Burke et al. [38] conducted several trials that evaluated St. Croix, Romanov and cross-bred ewes grazing tall fescue or bermudagrass pastures and did not observe any differences in pregnancy or lambing rates of the ewes due to pasture type. In contrast, Duckett et al. [7] reported a 4-d decrease in gestation length in Southdown ewes fed E+.

Lamb birth weight was lower in ewes consuming E+ in late pregnancy. The lower birth weight per ewe was similar to that previously reported in ewes of the same seed treatment and pregnancy terminated at d 133 [8,9]. These outcomes indicate that mycotoxin ingestion alters fetal growth during the time period of rapid fetal growth in the last trimester that are already apparent at d 133 of gestation and not due to any differences in gestation length. In twin-bearing ewes, around 80% of fetal growth occurs during the last trimester [50] and mycotoxin ingestion limits this [8,9].

Ergot alkaloid ingestion in last trimester reduced early milk production (d 1) by 40%. In contrast, E+ fed at any time reduced milk production on d 2 of lactation by 69% to 83% compared to E−/E−. Exposure to ergot alkaloids in second trimester reduced mid-lactation milk production by 50%. Together, these results indicate that mycotoxin ingestion reduces milk production in ewes. Milk production was moderately related with serum prolactin levels at d 50 (r = 0.30 with milk 21 d), 105 (r = 0.35 with milk d 1 and 2), 133 (r = 0.44 with milk d 1 and 2) and 145 (r = 0.47 with milk d 1 and 2). Similarly, others have reported reductions in milk production in cattle consuming E+ tall fescue pastures [51] and silage [33,52]. In contrast, Burke et al. [38] did not observe changes in milk yield, although reductions in milk fat were observed for cows grazing E+ than E− from d 57 to 180 d postpartum. In sheep, prolactin increases throughout gestation and initiates milk letdown prior to parturition [53,54]. Mammary development in sheep is complete by parturition [55] and the reduction in serum prolactin during gestation with E+ fescue may limit the capacity for milk production [56]. However, vasoconstriction may also influence lactation through restriction of blood flow to the udder [56,57].

Pre-weaning lamb growth rates and body weight change during pre-weaning growth was lower in lambs of E+ ewes fed in mid trimester. This is likely associated with the decrease in milk production in these ewes on d 21 of lactation. The correlations between ADG from birth to d 28 and milk production at d 1 (r = 0.30, *p* = 0.037), d 2 (r = 0.40, *p* = 0.0047) and d 21 (r = 0.25, *p* = 0.048) were moderate. The correlation between milk production on d 2 of lactation and overall ADG was 0.25. Weaning weights were reduced in lambs whose dams were fed E+. Similarly, others (3, 6) reported reduced pre-weaning growth rates and lower weaning weights in calves of cows that grazed E+ tall fescue compared to novel endophyte, E− or orchardgrass pastures. Burke et al. [38] did not observe any changes in birth or weaning weights of lambs born to St. Croix and St. Croix x Romanov dams that grazed E+ fescue. Others [58] found that low-birth-weight lambs remained lighter than controls from birth to 4 weeks of age; however, body weights were similar at 8 wk of age, indicating that there was some compensatory growth.

## 5. Conclusions

Feeding endophyte-infected tall fescue seed to ewes lowered serum prolactin and carotid luminal area, indicating the effectiveness of seed treatment to induce fescue toxicosis and vasoconstriction. Ergot alkaloid ingestion in the last trimester reduced lamb birth weight; however, ergot alkaloid exposure during pregnancy reduced milk production, pre-weaning growth rate and total weaning weight. These results suggest that ingestion of ergot alkaloids during pregnancy can negatively influence lactation, growth of offspring and birth and weaning weights.

## Figures and Tables

**Table 1:** Body weight change (BW), dry matter intake (DMI) and average daily gain (ADG) for ewes of different genotypes (DRD2 SNP) fed E− or E+ seed.

Ewe Performance	Maternal DRD2 Genotype			Fescue Seed MID		Fescue Seed LATE		Standard
	AA	AG	GG	E−	E+	E−	E+	Error (SE)
Ewes (n)	12	26	14	24	28	24	28	
BW change, kg								
d 28–85	5.23	2.94	5.07	5.10	3.72	4.93	3.90	4.32
d 86–136	20.67	19.00	16.68	19.00	18.56	21.34 ^c^	16.23 ^d^	6.01
d 28–136	26.68	22.81	22.76	25.48	22.69	27.87 ^a^	20.29 ^b^	6.61
DMI, kg/d								
d 28–85	1.93	1.94	1.88	1.91	1.92	1.94	1.89	0.080
d 86–136	2.69	2.72	2.64	2.67	2.69	2.67	2.69	0.24
d 28–136	2.35	2.37	2.29	2.32	2.35	2.35	2.33	0.15
ADG, g/d								
d 28–85	93.2	52.5	90.4	91.0	66.4	98.0	69.5	76.99
d 86–136	430.0	395.4	347.2	395.5	386.3	444.1 ^c^	337.7 ^d^	125.15
d 28–136	246.5	210.6	210.6	235.6	209.7	257.8 ^a^	187.6 ^b^	61.11

^a,b^ Means in the same row differ (*p* < 0.05) by fescue seed treatment fed during LATE gestation. ^c,d^ Means in the same row differ (*p* < 0.10) by fescue seed treatment fed during LATE gestation.

**Table 2:** Parturition characteristics in ewes of different genotypes (DRD2 SNP) fed E− or E+ seed.

Ewe Parturition Variables	Maternal DRD2 Genotype			Fescue Seed MID		Fescue Seed LATE		Standard Error
Per Ewe Basis	AA	AG	GG	E−	E+	E−	E+	SE
Ewes (n)	12	26	14	24	28	24	28	
Est. lamb no.	1.79	1.82	1.68	1.80	1.73	1.70	1.83	0.43
Gestation, d	145.5	145.0	146.3	145.0	146.2	146.5	144.7	2.67
Ewe difficulty	1.54	1.40	1.44	1.40	1.52	1.50	1.41	0.92
Lambs born	1.93	1.58	1.68	1.75	1.70	1.82	1.64	0.63
Lambs alive	1.94	1.55	1.36	1.74	1.50	1.65	1.58	0.72
Lamb vigor	1.87	1.89	2.08	2.01	1.88	2.02	1.87	0.75
Lamb sex ratio	1.44	1.46	1.48	1.59	1.33	1.48	1.44	0.45
Birth weight, kg	8.52	8.52	8.98	8.84	8.52	9.14 ^a^	8.21 ^b^	0.94
Crown-rump, cm	54.72	55.30	57.71	56.31	55.51	56.91	54.91	3.14

^a,b^ Means in the same row differ (*p* < 0.05) by fescue seed treatment fed during LATE gestation.

**Table 3:** Lamb pre-weaning growth by dam genotype, seed and stage of gestation.

Lamb Growth	Maternal DRD2 Genotype			Fescue Seed MID		Fescue Seed LATE		Standard Error
	AA	AG	GG	E−	E+	E−	E+	SE
Ewes (n)	12	26	14	24	28	24	28	
Lamb BW change, kg/ewe								
d 28	10.84 ^b^	14.19 ^a^	8.97 ^b^	12.31 ^g^	10.36 ^h^	11.82	10.85	2.88
d 56	11.35 ^c,d^	12.80 ^c^	9.75 ^d^	12.22 ^g^	10.38 ^h^	11.93	10.67	3.40
d 75 (wean)	6.64 ^d^	8.56 ^c^	7.90 ^d^	8.10	7.31	7.81	7.59	2.36
Weaning wt, kg/ewe	37.10 ^d^	43.74 ^c^	35.20 ^c,d^	41.25 ^e^	36.11 ^f^	40.32 ^i^	37.04 ^j^	5.30
Lamb ADG, g·ewe^−1^·d^−1^								
Birth to d 28	431.7 ^b^	535.8 ^a^	361.3 ^b^	485.3 ^e^	400.6 ^f^	469.2	425.8	120.5
d 28 to 56	395.0 ^a,b^	460.7 ^a^	347.4 ^b^	435.0 ^c^	367.1 ^d^	431.0	380.0	120.4
d 56 to 75	352.5	407.6	387.2	403.0	361.8	397.4	367.4	122.6
Overall	389.7 ^b^	466.8 ^a^	360.4 ^b^	438.5 ^e^	372.7 ^f^	423.8	387.5	71.4

^a,b^ Means in the same row with uncommon superscripts differ (*p* < 0.05) by genotype. ^c,d^ Means in the same row with uncommon superscripts differ (*p* < 0.10) by genotype. ^e,f^ Means in the same row with uncommon superscripts differ (*p* < 0.05) by fescue seed treatment fed during MID gestation. ^g,h^ Means in the same row with uncommon superscripts differ (*p* < 0.10) by fescue seed treatment fed during MID gestation. ^i,j^ Means in the same row with uncommon superscripts differ (*p* < 0.10) by fescue seed treatment fed during LATE gestation.
